# Monitoring the Cytoskeletal EGF Response in Live Gastric Carcinoma Cells

**DOI:** 10.1371/journal.pone.0045280

**Published:** 2012-09-27

**Authors:** Marco Felkl, Kazmar Tomas, Matej Smid, Julian Mattes, Reinhard Windoffer, Rudolf E. Leube

**Affiliations:** 1 Institute of Molecular and Cellular Anatomy, RWTH Aachen University, Aachen, Germany; 2 Software Competence Center Hagenberg GmbH, Hagenberg, Austria; Dresden University of Technology, Germany

## Abstract

Altered cell motility is considered to be a key factor in determining tumor invasion and metastasis. Epidermal growth factor (EGF) signaling has been implicated in this process by affecting cytoskeletal organization and dynamics in multiple ways. To sort the temporal and spatial regulation of EGF-dependent cytoskeletal re-organization in relation to a cell’s motile behavior time-lapse microscopy was performed on EGF-responsive gastric carcinoma-derived MKN1 cells co-expressing different fluorescently labeled cytoskeletal filaments and focal adhesion components in various combinations. The experiments showed that EGF almost instantaneously induces a considerable increase in membrane ruffling and lamellipodial activity that can be inhibited by Cetuximab EGF receptor antibodies and is not elicited in non-responsive gastric carcinoma Hs746T cells. The transient cell extensions are rich in actin but lack microtubules and keratin intermediate filaments. We show that this EGF-induced increase in membrane motility can be measured by a simple image processing routine. Microtubule plus-ends subsequently invade growing cell extensions, which start to accumulate focal complexes at the lamellipodium-lamellum junction. Such paxillin-positive complexes mature into focal adhesions by tyrosine phosphorylation and recruitment of zyxin. These adhesions then serve as nucleation sites for keratin filaments which are used to enlarge the neighboring peripheral keratin network. Focal adhesions are either disassembled or give rise to stable zyxin-rich fibrillar adhesions which disassemble in the presence of EGF to support formation of new focal adhesion sites in the cell periphery. Taken together the results serve as a basis for modeling the early cytoskeletal EGF response as a tightly coordinated and step-wise process which is relevant for the prediction of the effectiveness of anti-EGF receptor-based tumor therapy.

## Introduction

The epidermal growth factor (EGF) has profound effects on proliferation, differentiation and cellular motile behavior [1], [2], [3]. These pleiotropic effects rely on activation of the cognate cell surface EGF receptor (EGFR), which dimerizes upon ligand binding. Concomitant activation of the intracellular kinase domain leads to autophosphorylation of tyrosine residues within the cytoplasmic domain of the receptor to which various adaptors and signaling molecules are recruited.

The rationale for treatment attempts of various tumor types with antibodies that are directed against the EGFR is based on the observation that disturbance of EGFR signaling is linked to malignant transformation and tumor progression [3], [4], [5]. Despite the success of this approach in some instances differences in responsiveness have been major challenges in selecting tumors that are suitable for antibody treatment [6]. The molecular basis of the differential EGF response has been addressed in several studies focusing primarily on the regulation of cell proliferation [7]. The invasion-blocking effects of EGFR antibodies, however, appear to rely on fundamentally different mechanisms affecting cell motility [8], [9]. It has been reported that EGF induces changes in cell shape, membrane ruffling and lamellipodia formation eventually leading to increased cell migration [10], [11]. These alterations have been attributed to re-organization of the three major cyotskeletal filament systems comprising the actin-based microfilaments, the tubulin-containing microtubules and the intermediate filaments that are composed of keratin polypeptides in epithelial cells [12], [13], [14], [15], [16], [17], [18], [19]. In addition, extracellular matrix (ECM) adhesion sites of variable size and molecular complexity have been identified as key players in determining EGF-dependent motile cell behavior [20], [21], [22], [23]. Despite the wealth of available data and observations it is still not fully understood, precisely how and in which way the re-organization of the different filament systems and the associated adhesion structures are sequentially and spatially organized and coordinated in EGF-treated cells. The ongoing challenge is to precisely sort out and identify the successive steps of the motile EGF response as a basis for the elucidation of the molecular mechanisms that regulate the multiple decisions needed to initiate and execute the complex program of directed cell motility.

The goal of the current study was therefore to assess the temporal and spatial coordination of cytoskeletal filament re-organization and focal ECM adhesion dynamics in response to EGF. This was done by single, double and even triple labeling of cells for the investigation of EGF-induced re-organization in live cells by time-lapse fluorescence microscopy in combination with differential interference contrast (DIC) imaging. In this way parameters were derived to identify distinct and successive steps in the early EGF response. This will help to qualitatively and quantitatively assess EGF responsiveness of tumor cells at very high precision.

## Materials and Methods

### DNA Cloning

To label cytoskeletal filaments cDNAs encoding mRFP-actin [24], HK18-YFP [25], and EB3-CFP (plasmid 887) were used. EB3-CFP-encoding plasmid 887 was generated by inserting an EcoRI-BamHI fragment amplified by PCR from an EB3-GFP-encoding plasmid vector (kindly provided by Dr. Duden) with primers 10-01 (CCG AAT TCA TGG CCG TCA ATG TGT AC) and 10-02 (TTG GAT CCC AGT ACT CGT CCT GGT CTT C) into vector pECFP-N1 (Clontech Laboratories). For simultaneous labeling of actin and keratin filaments, a multicistronic vector system was prepared. To this end, a PCR-amplified (primers 09-44 TCA AGC TTA TGG CCT CCT CCG AGG AC and 09-45 CGA CTA GTC TAG AAG CAT TTG CGG TG) HindIII-SpeI mRFP-actin coding fragment [24] was cloned into the modified bicistronic vector 883 pMC-ECFP-H-N (kindly obtained from Dr. Christian Mielke; [26]) resulting in construct 884. In addition, a PCR-amplified (primers 09-46 CGA AGC TTA TGA GCT TCA CCA CTC GC and 09-47 CCG AAT TCT TAC TTG TAC AGC TCG TC) HindIII-EcoRI human keratin 18-EYFP-coding fragment was inserted into biscistronic pMC-derivative 880 pMC-EYFP-P (also from Dr. Mielke) resulting in vector 885. Subsequently, the ClaI-NotI-fragment of 884 encompassing the CMV promoter-mRFP-actin-IRES segments was cloned into the ClaI-NotI sites of plasmid 885 resulting in vector construct 886. This vector supports the coupled production of the actin- and keratin 18-fusion proteins together with the puromycin selection marker from the same CMV promoter in transfected cultured cells.

Construct paxillin-pDsRed2-N1 coding for fusion protein paxillin-dsRed was kindly provided by Dr. Horwitz [27], a RFP-zyxin-encoding construct was obtained from Dr. Huttenlocher [28], and expression construct pEYFP-N1 was from Clontech (Mountain View, CA). The constructs CFP-dSH2 and YFP-dSH2 were given to us by Dr. Geiger [29], [30].

### Cell Culture

Human gastric cancer-derived MKN1 cells were obtained from the Cell Bank RIKEN BioResource Center (Tsukuba, Japan) via Dr. Luber [31], [32]. They were maintained in RPMI 1640 medium (Invitrogen/Gibco, Darmstadt, Germany) with 2 mM L-glutamine (Invitrogen/Gibco) and 10% Sera Plus fetal bovine serum (PAN Biotech, Aidenbach, Germany) in a humidified incubator at 37°C with 5% CO_2_. Human gastric carcinoma-derived Hs746T were obtained from the ATCC Cell Biology Collection (LGC Standards GmbH, Wesel, Germany) through Dr. Luber [32]. They were grown in Dulbecco’s Modified Eagle Medium (MEM, Sigma Aldrich Chemie GmbH, Steinheim, Germany) with GlutaMAX I (Invitrogen/Gibco) and 10% fetal bovine serum (PAN Biotech) at 37°C and 5% CO_2_.

DNA of suitable expression constructs was transfected into cultured cells with the help of Lipofectamine 2000 reagent following the instructions provided by the manufacturer (Invitrogen). Microscopic analyses ensued 48 h later. To this end, cells were transferred from standard culture medium into phenol red-free Hanks’ medium (containing Hanks’ salt solution, 25 mM Hepes, MEM non-essential amino acid solution and MEM amino acid solution (all from Invitrogen/Gibco), 5% fetal bovine serum (PAN Biotech), 4.8 mM N-acetyl-L-cysteine (Sigma), pH 7.45) and placed in a microscope chamber at 37°C and ambient air.

For assessment of drug-induced alterations of cytoskeletal and focal adhesions dynamics, cells were seeded in glass bottom Petri dishes (MatTek Corporation, Ashland, MA) coated with 100 µg/ml rat tail collagen type I (BD Biosciences, Bedford, MA) and grown under standard culture conditions for 48 h. After transfer into imaging medium they were moved to the microscope chamber and were treated after short preincubation periods with 5–100 ng/ml epidermal growth factor (EGF) (E9644 from Sigma, München, Germany) in phenol red-free Hanks’ medium. Of note, the effective EGF concentration in the medium could not be precisely controlled, because the serum samples used during the preincubation period contained slightly different EGF concentrations and prolonged EGF storage reduced its activity. Therefore, EGF was routinely added at 5 ng/ml and 30 ng/ml in each set of experiments. For quantitative determinations and direct comparisons, however, identical concentrations of the same aliquots were used in parallel to exclude differences in EGF concentrations. In some experiments the EGFR antibody cetuximab (Erbitux, Merck Serono GmbH, Darmstadt, Germany) was used at 1 µg/ml.

### Imaging and Image Analysis

Fluorescence and differential interference (DIC) images were recorded at 16 bit with an inverse confocal laser scanning microscope (Zeiss LSM 710) using a Plan-Apochromat 63×/1.40 oil DIC M27 objective (Zeiss) and the ZEN 2009 software. To quantitatively detect enhanced yellow fluorescent protein (YFP) in the cytoplasm the 514 nm argon laser line (Ar-Laser Multiline 458/488/514 nm; Zeiss) was used to record images at a resolution of 1024×1024 pixel with a pinhole setting of 1 airy unit (AU) and beam splitter MBS 458/514 (Zeiss). For detection of multiple fluorophores the respective fluorescence was recorded in separate tracks to minimize bleed through. One track was used for YFP (514 nm argon laser line; beam splitter MBS 458/514), another track for simultaneous detection of monomeric red fluorescent protein (mRFP) or DsRed2 (543 nm helium-neon laser line [Laser HeNe 543 nm, Zeiss]) and enhanced cyan fluorescent protein (CFP) (458 nm argon laser line; beam splitter MBS 458/543 for both). Laser intensity and resolution were adjusted in each instance to maintain sufficient image quality while avoiding photobleaching as much as possible. In some instances, DIC images were recorded in parallel.

Data were exported and further processed using ImagePro Plus (Media Cybernetics), Fiji (http://fiji.sc/wiki/index.php/Fiji) and Photoshop CS3 (Adobe) to prepare composite figures and videos that were converted into mpeg using the TMPG encoder (http://www.tmpgenc.net). For semi-automated analysis of the microscopic records and for the purpose of motion quantitation [33] custom-built C++ and python software routines were developed.

## Results

### Detecting and Measuring EGF-induced Ruffling in Live Cells

Gastric carcinoma-derived MKN1 cells were chosen to examine the EGF response because of their amenable morphology, detailed characterization, widespread use and high level EGF receptor expression [34], [35]. Furthermore, this cell line was recently shown to respond to EGF treatment by increased EGFR phosphorylation that could be inhibited by EGFR antibodies [31]. By time-lapse recording of DIC images we now observed that addition of EGF leads to increased ruffling/lamellipodial activity within 1–2 min. Cell surfaces with multiple ruffles gave rise to protruding lamella at the cell-ECM interface of the leading edge (e.g., Videos S1, S2).

**Figure 1:**
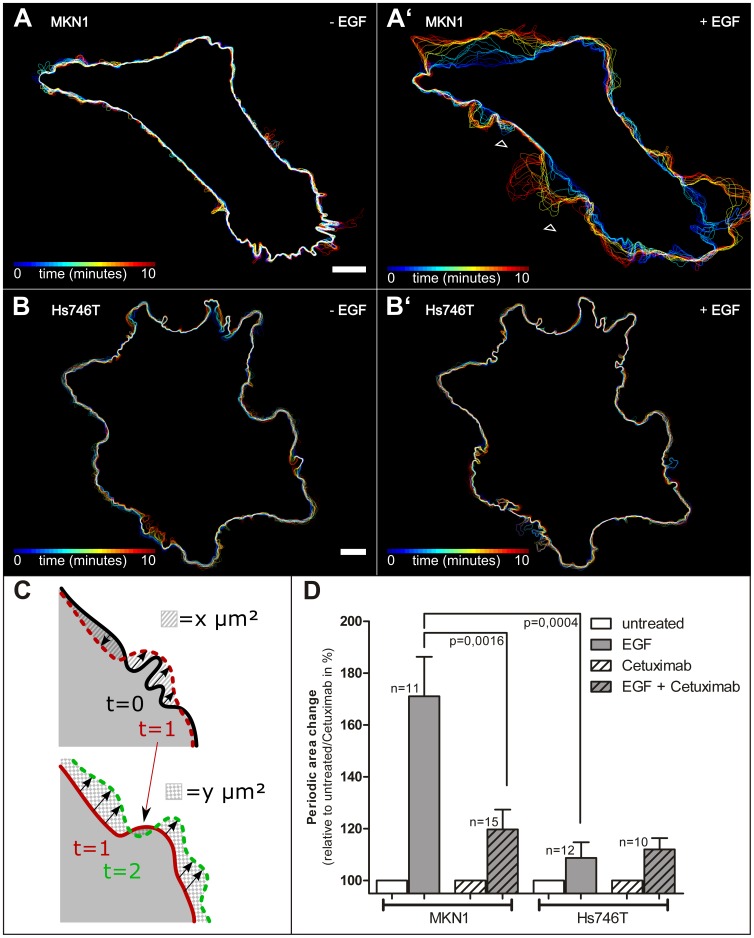
EGF induces ruffling in MKN1 responder cells. MKN1 and Hs746T cells were transfected to produce enhanced yellow fluorescence protein (YFP) under CMV-promoter control. The fluorescence was recorded by confocal laser scanning microscopy three times per minute for 10 min in the absence of EGF and 10 min after addition of EGF. (A, B) show the color-coded cell contour changes in the absence of EGF, (A’, B’) after addition of 5 ng/ml EGF. The data are derived from Video S1. Bar, 10 µm. The arrowheads indicate places where the wavelike movement of the cell border during ruffling can be discovered from the sequence of cell contours in MKN1 cells in the presence of EGF (compare A, A’). Note that such changes are not observed in Hs746T cells upon EGF treatment (B, B’). (C) Illustration of changes in cell contour for three time points to elucidate the measuring procedure of the periodic area change Δ_P_A. The cell contour recorded at t = 0 is shown in black at top, at t = 1 in red (dashed line at top, solid line at bottom), and at t = 2 in green. The changes in cell area are highlighted in top by a striated area for the comparison of t = 0/t = 1 leading to |Δ_1_| and below by checkered pattern for the comparison of t = 1/t = 2 (|Δ_2_|). (D) Quantitative assessment of dynamic cell size changes in response to EGF. The histogram represents the periodic area change as measured from time-lapse series of fluorescence micrographs taken from MKN1 and Hs746T cells that had been labeled with YFP. Plotted is the relative increase in % of the measured value with respect to this value before addition of EGF (respectively for untreated and Cetuximab treated cells). Each set of measurements refers to a 10 min observation period with a recording frequency of 3 images/min. Cells were monitored in the absence of EGF (“untreated”; defined as 100%), in the presence of 5 ng/ml EGF (“EGF”), in the presence of 1 µg/ml Cetuximab (“Cetuximab”) and in the presence of both 5 ng/ml EGF and 1 µg/ml Cetuximab (“EGF + Cetuximab”). Note the increased EGF-dependent and Cetuximab-inhibitable ruffling activity (p-value determined by Student’s t-test) in MKN1 cells that is not discernible in Hs746T cells.

For a precise quantitative assessment of the rapidly alternating cycles of protrusion and retraction of ruffles and lamellipodia in EGF-treated MKN1 cells a combined experimental and computational method was developed. To this end, cells were fluorescently labeled by transfection with an YFP-encoding expression construct. Images were recorded at a frequency of 3/min in the absence and presence of EGF. In this way the exact contours of the cells could be detected by fluorescence microscopy and periodic peripheral cell changes could be measured according to the following protocol: For each two consecutive points in time t_i-1_ and t_i_, (i = 1, …, N) corresponding to two consecutive frames in the recorded microscopic film, the area change |Δ_i_| is measured as illustrated in Fig. 1C, where the striated and checkered patterned areas (as positive values) correspond to |Δ_1_| and |Δ_2_|, respectively. The *periodic area change* denoted by Δ_P_A is then quantified as the sum over the area changes |Δ_i_| for all pairs of consecutive frames – corresponding to consecutive points in time (t_0_, t_1_), …, (t_i−1_, t_i_), …, (t_N−1_, t_N_) – of the film, divided by the elapsed time t_N_−t_0_. Video S1 shows a typical recording before and after EGF addition, which induced cell spreading, appearance of lamellipodia, filopodia and lamella as well as periodic wavelike formation of multiple ruffles. The increased plasma membrane motility after addition of EGF is readily apparent in the derived color-coded overlays of cell contours (compare Fig. 1A and A’). Using this approach, the EGF response was measured in 11 cells following a standard protocol: Cells were first imaged for 10 min, 5 ng/ml EGF were then added between 10 and 12 min, and another 10 min recording (12 min–21 min) ensued. The results of the quantification are depicted in Fig. 1D. The histogram highlights the dramatic increase in ruffling activity in MKN1 cells upon EGF treatment. This response was inhibited to a large degree by inhibitory Cetuximab EGFR antibodies (n = 15). To further explore the usefulness of this protocol gastric carcinoma-derived Hs746T cells were examined which are known to be non-responsive to EGF [32]. Fig. 1 B, B’, D demonstrate that EGF treatment did not lead to a Cetuximab-inhibitable increase in ruffling (n = 12 for EGF alone; n = 10 for Cetuximab and EGF). Taken together, the results show that this comparatively simple procedure is suitable to precisely measure specific short-term effects of EGF on cell motility and to distinguish between EGF-responding and non-responding tumor cells.

### Cytoskeletal Network Rearrangements in Response to EGF

To directly monitor the EGF response of the different filamentous cytoskeletal systems, expression constructs for fluorescent protein reporters were prepared and introduced into MKN1 cells.

**Figure 2:**
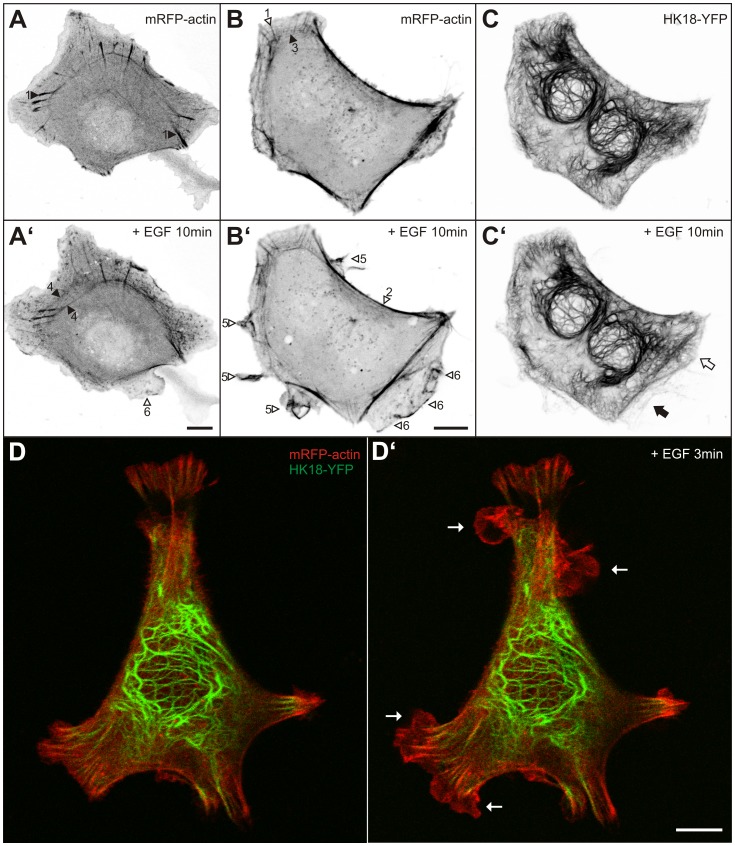
EGF induces actin-rich and keratin-free ruffles and lamellopodia. (A–B’) Fluorescence microscopy of MKN1 cells producing mRFP-actin before (A, B) and 10 min after addition of 30 ng/ml EGF (A’, B’). Focal adhesion-associated stress fibers (1), submembranous stress fibers (2), transverse fibers (3) and transverse dorsal arcs giving rise to centrally located circular bundles (4) can be distinguished. In addition, cortical enrichment of actin is noticeable in ruffles and lamellipodia (5) and in lamellar extensions (6). Note that prominent actin stress fibers persist, actin-rich ruffles, lamellipodia, and lamella are generated, and that circular actin bundles translocate toward the cell center in the presence of EGF. (C–C’) The binucleated cell shown in (B, B’) also expressed HK18-YFP. Note that only the older lamella is positive for HK18-YFP fluorescence (white arrow in C’) whereas the newly-formed lamella below is not (black arrow in C’). (D–D’) The overlay images show a live MKN1 cell that is labeled with HK18-YFP (green) and mRFP-actin (red) before (D) and 3 min after addition of 5 ng/ml EGF (D’; complete sequence in Video S2). Note again the appearance of multiple keratin-free ruffles (arrows) upon EGF treatment. Bars, 10 µm.

The actin system was analyzed by introducing an mRFP-actin-encoding gene construct. The different types of actin fibers were clearly delineated in transfected living cells (n = 16). Typical examples are depicted in Fig. 2A–B’ (see also [36], [37], [38]): Most prominent were focal adhesion-anchored stress fibers that extend from the cell centre towards the periphery (1 in Fig. 2A, B). Prominent were also actin stress fibers that are in close proximity to the plasma membrane (2 in Fig. 2B’). Less prominent were peripheral transverse fibers, which are oriented in parallel to the plasma membrane and interconnect adjacent focal adhesions (3 in Fig. 2B). Occasionally, transverse dorsal arcs giving rise to circular bundles were noted that are located more centrally (4 in Fig. 2A’). The examples presented in Fig. 2A–B’, D–D’ and Video S2 illustrate how actin distribution and dynamics changed in response to EGF (total number of cells examined by time-lapse fluorescence imaging  = 16) and how this correlated with altered membrane morphology and motility. Multiple actin-containing ruffles appeared almost instantaneously after EGF supplementation (arrowheads #5 in Fig. 2B’; Video S2). They were highly dynamic and propagated along the cell surface. Extensive lamella (arrowheads #6 in Fig. 2A’, B’; Video S2) appeared within the next 5 min. Pre-existing actin stress fibers persisted after EGF addition (Fig. 2A’). Moreover, an increase of actin stress fibers was observed in 50% of exposed cells. Particularly noticeable was the inward movement of circular actin stress bundles in several cells (Fig. 2A, A’).

By transfection of cells with a polycistronic vector coding for mRFP-actin and human keratin 18 fused to EYFP (HK18-YFP) simultaneous monitoring of the actin and intermediate filament cytoskeletons was accomplished (n = 16; representative examples depicted in Fig. 2C–D’). These experiments showed that the organization of the keratin network was not immediately affected by EGF treatment. The multiple ruffles and lamellipodia that appeared upon EGF addition were devoid of keratin-filaments (arrows in Fig. 2D’; Video S2).

**Figure 3:**
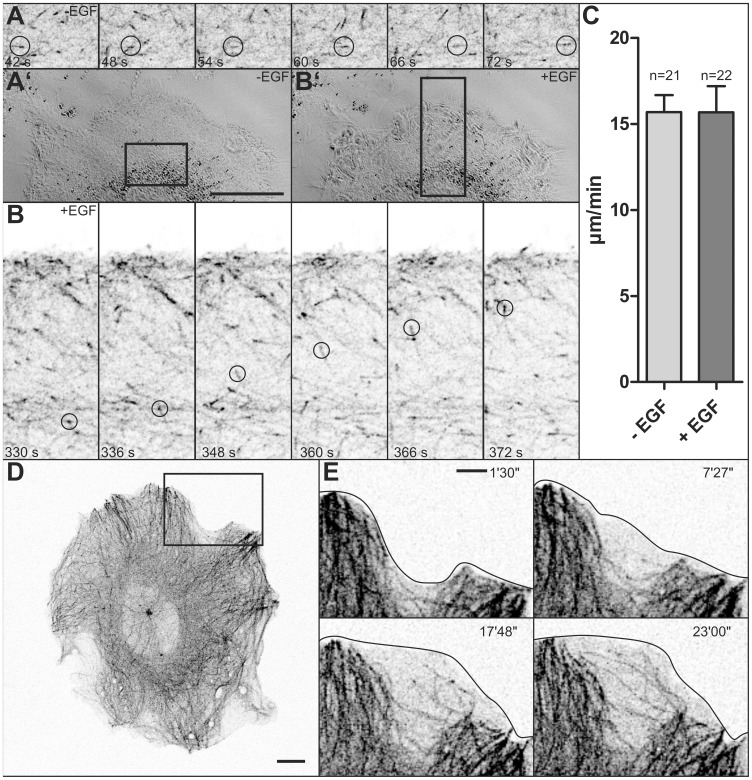
EGF does not affect microtubule plus-end-dynamics but leads to exploratory microtubule growth into newly-formed cell extensions. Microtubules were labeled by EB3-CFP in MKN1 cells. (A, B) The images are taken from Video S3. They show DIC images (A’ at time point 42 s, B’ at time point 330 s) and details of fluorescence (inverse presentation in A, B; corresponding regions marked in A’, B’) of a cell that was treated with 30 ng/ml EGF from 192 s onwards. Examples of growing microtubule ends are circled in A, B. The histogram in (C) depicts the mean growth rates ± SEM of individual microtubules before (−EGF) and after addition of EGF (+EGF) in 7 MKN1 cells (number of plus-ends examined was 21 for -EGF and 22 for +EGF (n = 5 for 100 ng/ml and n = 17 for 30 ng/ml). (D–E) Present fluorescence micrographs (inverse presentation) of a MKN1 cell synthesizing EB3-CFP prior to addition of 100 ng/ml EGF. Micrographs in (E) are taken from Video S4 and show a peripheral region of the cell depicted in D (marked by square), where a stable lamellum is formed upon EGF addition (times after EGF supplementation in upper right corner). Stages of microtubule extension into the newly-formed lamellum are depicted. Bars, 5 µm in A’ (same magnification in B’), E; 10 µm in D.

**Figure 4:**
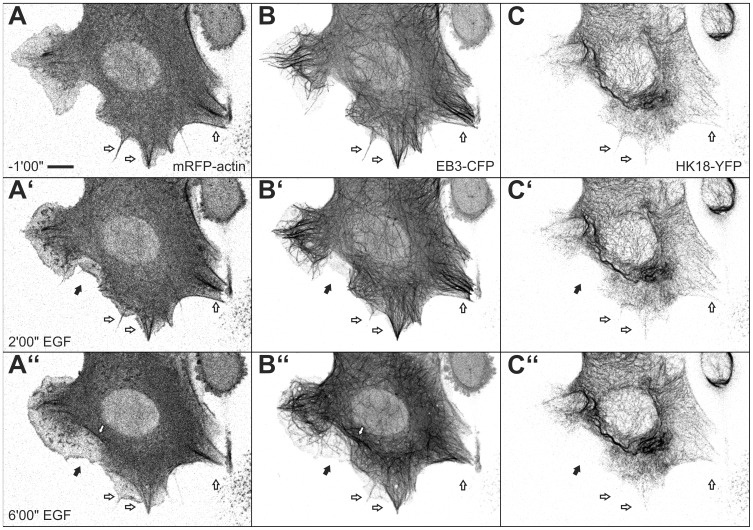
Triple labeling of MKN1 cells reveals sequential rearrangement of cytoskeletal filaments in response to EGF. MKN1 cells were co-transfected with expression constructs for mRFP-actin (A, A’, A”), EB3-CFP (B, B’, B”) and HK18-YFP (C, C’, C”) and were imaged before and after addition of 30 ng/ml EGF. Note that EGF-induced cell extensions are positive for actin and only later recruit microtubules (black arrows). These areas are still negative for keratin after 6 min of EGF treatment. Note also the co-alignment of actin bundles with microtubules (white arrows). A time-lapse sequence of the same cell beginning 10 min after EGF addition is provided as Video S5. Bar, 10 µm.

To examine the effect of EGF on microtubule dynamics, microtubules were labeled with a construct coding for the plus-end binding protein EB3 fused to enhanced cyan fluorescent protein (EB3-CFP), which was a superior alternative to labeling with fluorescent tubulin fusion proteins. Low expression of the transgene resulted in predominant labeling of microtubule plus-ends (Fig. 3A, B; Video S3) whereas overexpression resulted in labeling of the entire microtubule cytoskeleton (Figs. 3D, E, 4B–B”; Videos S4, S5). To assess the influence of EGF on overall microtubule dynamics, EB3-CFP-labeled plus-ends were individually tracked by hand. A typical experiment is depicted in Fig. 3A, B and corresponding Video S3 in which multiple, rapidly growing microtubule plus-ends are detectable. These analyses revealed that EGF treatment does not affect the speed of microtubule growth (compare Fig. 3A and 3B; see also Video S3). Quantification showed that the plus-ends moved at an average speed of 15.69±0.98 µm/min in the absence of EGF (21 plus-ends in 5 cells) and that addition of EGF did not alter that (15.68±1.52 µm/min, 22 plus-ends in 4 of the 5 cells that were also examined before EGF addition; P = 0.995 (Student’s t-test; Fig. 3C). Of note, EGF concentration did not affect the results, since 17 measurements were done in cells at 30 ng/ml EGF and 5 measurements in cells at saturating EGF concentrations (100 ng/ml). Next, microtubule dynamics were analyzed in EGF-induced cell extensions. It was observed that newly-formed ruffles and lamellipodia were completely devoid of microtubules (Fig. 3D, E; Video S4). After several minutes microtubules emerged from the adjacent microtubule-rich cytoplasm and entered the lamellipodial/lamellar extensions. These microtubules traversed the space in a rather uncoordinated fashion. They often approached the plasma membrane resulting in deflection of the growing microtubule ends.

The DIC images provided in Video S3 further highlight other aspects of the EGF-induced migratory response presenting the hallmark features of migratory cells [37], [39]. Polarization of the cell becomes rapidly apparent after EGF addition by appearance of multiple ruffles at the leading edge. This is accompanied by cell contraction and formation of slender retraction fibers at the trailing edge upon translocation of the cell body. Translocation is coupled to rapid release of actin filaments and keratin filaments from the rear part of cells as can be seen in Video S2 (lower right). These observations support the conclusion that EGF treatment induces the canonical cytoskeleton-driven migratory response in MKN1 cells.

**Figure 5:**
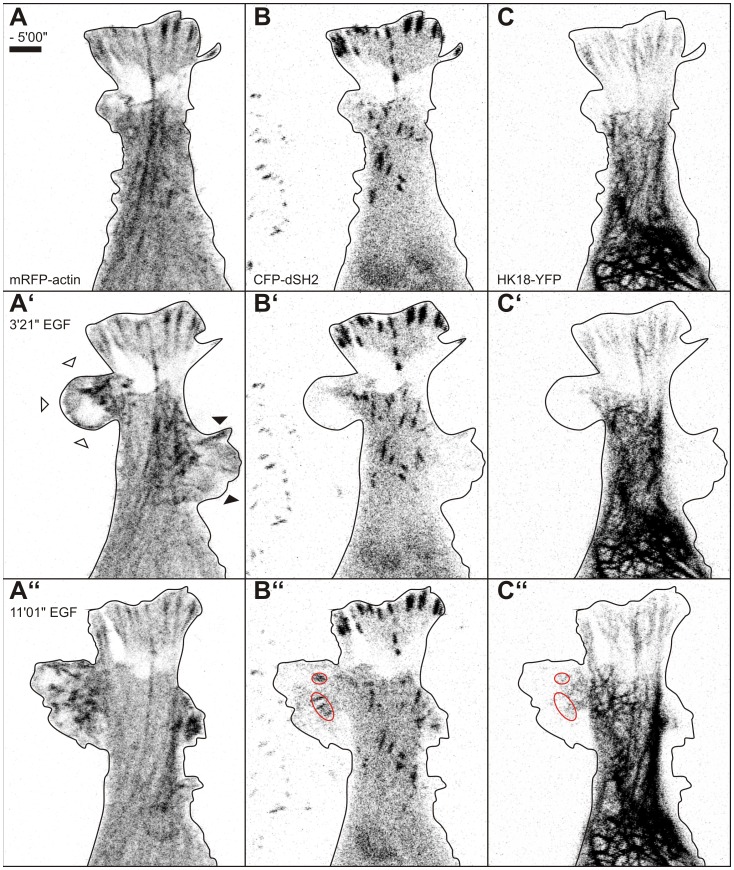
EGF evokes restructuring of the actin and keratin cytoskeleton in relation to ruffling, lamella formation and focal adhesion formation. The images show a recording of part of a MKN1 cell (see also Fig. 2, Video S2) that is labeled with mRFP-actin (left), CFP-dSH2 (middle) and HK18-YFP (right). The images show the fluorescence patterns 5 min before EGF addition (top) and 3 min 21 s (middle) and 11 min 1 s (bottom) after addition of 5 ng/ml EGF (complete sequence in Video S6). Note the appearance of actin-rich ruffles that either lead to lamella formation (white arrowheads in A’) as evidenced by dSH2-labelling (red ovals in B”) and extending keratin filaments (red ovals in C:”) or disappear without establishing focal adhesion-mediated contact to the extracellular matrix (black arrowheads in A’). Bar, 5 µm.

To directly correlate and delineate the temporal and spatial EGF response of all three cytoskeletal filament systems with altered cell motility, triple transfectants were examined by time-lapse fluorescence microscopy and parallel recording of DIC images (n = 3). These experiments revealed the successive and interdependent nature of cytoskeletal filament rearrangement as exemplified in Fig. 4 and Video S5. An increase of actin-rich but microtubule- and keratin-free ruffles and lamellipodia giving rise to lamella was first observed upon EGF addition (black arrows in Fig. 4A’, B’,C’). Subsequently, microtubules invaded these cell extensions (Fig. 4B”) but keratin-positive structures were not visible in these regions (black arrowhead in Fig. 4C”). At the same time the number of actin stress fibers increased gradually which aligned with microtubules (white arrows inside the cell depicted in Fig. 4A”, B”). Extension of the keratin network occurred last and was noted in large cytoplasmic extensions, which are presumably anchored to the extracellular matrix by stable adhesions (Fig. 5; Video S6).

### EGF-dependent Dynamics of Extracellular Matrix Attachment Sites in Relation to Cytoskeletal Organization

Adhesions to the extracellular matrix serve as attachment sites for actin filaments, they cross talk with microtubules and favor keratin filament formation (reviews in [37], [39], [40], [41], [42]). They first appear as nascent adhesions in newly-formed lamellipodia and transform into focal complexes at the lamellipodium-lamella transition maturing subsequently into focal adhesions, which either disassemble in motile cells or further mature into fibrillar adhesions in stably anchored cells [37], [39], [41]. We therefore wanted to examine the relationship between the EGF-induced alterations in cell motility and cytoskeletal organization with the dynamics of focal adhesions. To this end, time-lapse recordings of multiply-labeled cells were carried out.

Extracellular matrix attachment sites were detected in live MKN1 cells using either paxillin-DsRed (n = 12) or cyan fluorescent protein-coupled and duplicated SH2-domains of the pp60src kinase (n = 15), which were previously shown to bind to focal adhesion-enriched phosphotyrosine residues [30] with a preference for phosphorylated paxillin, CAS and FAK [29]. First, focal adhesion dynamics were correlated with actin and keratin organization (n = 5; example in Fig. 5 and corresponding Video S6). The actin-rich and keratin-lacking ruffles and active lamellipodia appearing immediately after EGF addition did not contain visible focal adhesion complexes (arrowheads in Fig. 5A’). These protrusions either disappeared or stabilized (compare Fig. 5A’–C’ with Fig. 5A”, B”, C”; Video S6). In the latter case, lamella formed under the motile membrane domains and focal adhesion-like structures emerged as tiny dots at the resolution limit that subsequently enlarged into elongated entities (circled in red in Fig. 5B”; Video S6). Keratin filament formation was initiated in the vicinity of these newly-formed focal adhesions resulting in peripheral network extension (circled in red in Fig. 5C”). Large (several µm) and stable adhesions such as those depicted at the top of the cell shown in Fig. 5 then served as attachment sites for actin stress fibers.

**Figure 6:**
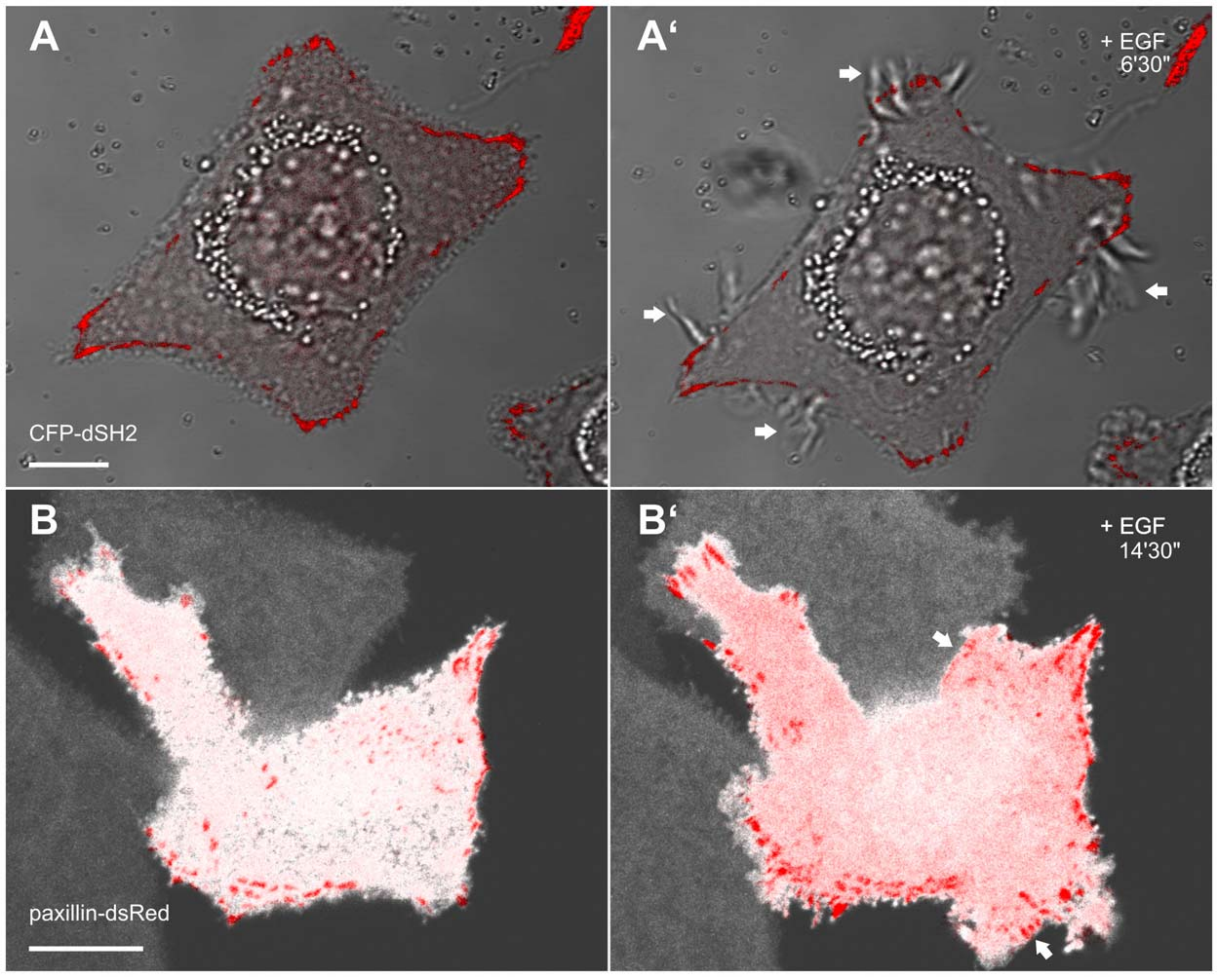
Lamella differ from ruffles by the presence of focal adhesions. The images shown in A, A’ are taken from Video S7 and present overlays of DIC recordings and CFP-dSH2 fluorescence microscopy (false red color) in MKN1 cells. Note the induction of abundant, dSH2-negative ruffles (arrows) upon addition of 5 ng/ml EGF. The micrographs in B, B’ are taken from Video S8 that shows the overlays of YFP- (white) and paxillin-dsRed-fluorescence in a MKN1 cell before (B) and after addition of EGF (5 ng/ml). Formation and turnover of paxillin-positive focal adhesions are detectable in regions of newly-forming lamella (arrows). Bars, 10 µm.

These observations suggested that ruffling and lamellipodial activity occur prior to the establishment of detectable focal adhesions. To further investigate this, focal adhesion dynamics were correlated with membrane dynamics (n = 27). The corresponding fluorescence images and DIC recordings of a live MKN1 cell depicted in Fig. 6A, A’ and Video S7 demonstrate that ruffles (arrows in Fig. 6A’) do not contain focal adhesions or focal complexes and can co-exist for a long time with stable focal adhesions in close proximity. In another experimental setup MKN1 cells were double labeled with YFP as cytoplasmic marker and paxillin-dsRed as a focal adhesion marker (n = 8; example in Fig. 6B, B’ and corresponding Video S8). The depicted sequence shows the appearance of novel paxillin-labeled focal adhesions after EGF treatment. They were formed in the cell periphery at the time, when lamellipodial activity subsided and stable lamella were generated (e.g., arrows in Fig. 6B’). Taken together, new focal contacts were formed within 30 min after EGF application in 78% of cells (n = 27) indicating that this is a further step in the pre-migratory phase.

**Figure 7:**
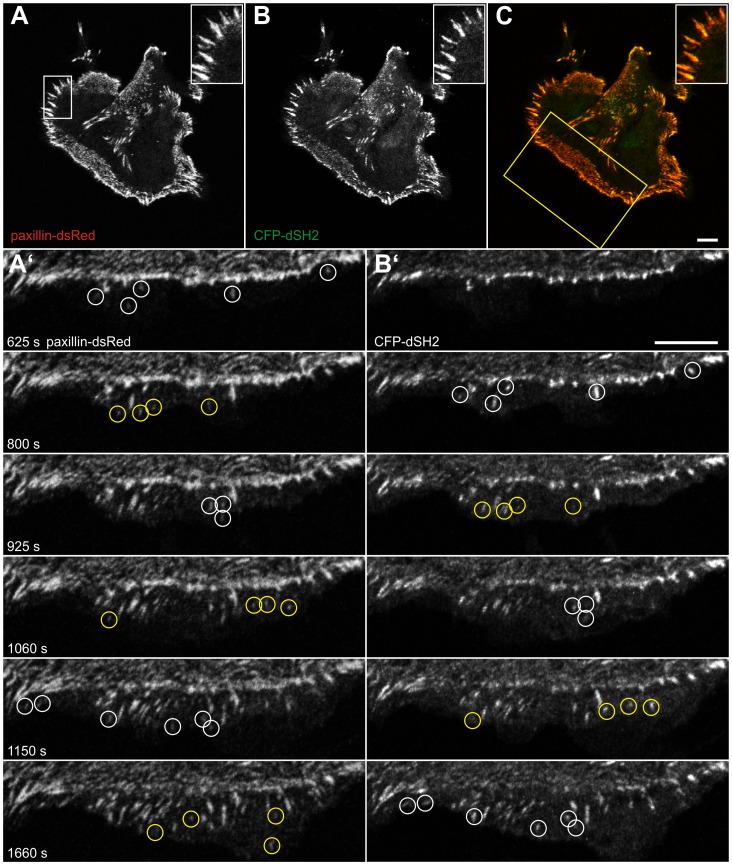
Paxillin is recruited to focal adhesions prior to tyrosine phosphorylation in newly-formed lamella upon EGF treatment. The images are taken from a MKN1 cell simultaneously expressing paxillin-dsRed (A, A’) and CFP-dSH2 (B, B’) in the absence of EGF (A–C; merged color images in C [CFP-dSH2 in false green color]) and after addition of 5 ng/ml EGF (A’, B’). The inserts in A–C depict the high degree of co-localization in the region delineated by white box in A at higher magnification. The images in (A’, B’) are taken from Video S9 (corresponding area demarcated by yellow box in C) demonstrating the appearance of paxillin-positive focal adhesion sites prior to dSH2 recruitment in a newly-forming lamellum (examples are highlighted by color coded circles in A’, B’). Bars, 10 µm.

**Figure 8:**
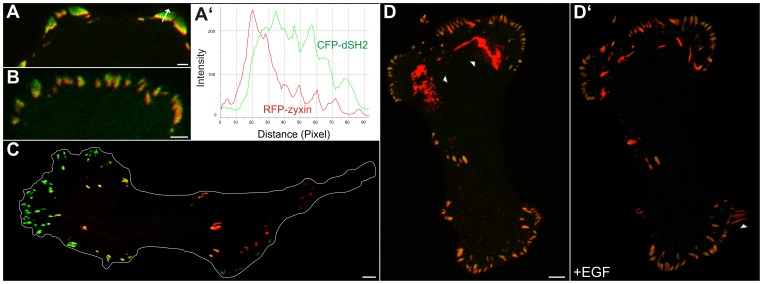
Zyxin partially co-distributes with dSH2 in focal adhesions and is recruited from zyxin-enriched fibrillar adhesion upon EGF treatment. MKN1 cells were co-transfected with constructs encoding RFP-zyxin and CFP-dSH2 (depicted in false green color). Note that both co-localize only partially in the more central parts of focal adhesions in the cell periphery (A, B). This is further highlighted by the line diagrams in A’ that were recorded along the arrow in A. In contrast, more centrally located focal adhesions and large fibrillar adhesions are predominantly positive for zyxin and contain very little dSH2 label (C, D). (D, D’) is taken from Video S10 and depicts a cell immediately before and 59 min after addition of 5 ng/ml EGF. Note the reduction of the very large zyxin-positive plaque-like areas (arrowheads in D) and the appearance of novel contacts in newly-formed lamella (arrowhead in D’). Bars, 2 µm in A, B; 5 µm in C, D.

In the next set of experiments different focal adhesion markers were co-expressed to distinguish different stages of EGF-induced focal adhesion formation and maturation. Direct comparison of the early focal adhesion marker paxillin-DsRed with CFP-dSH2 (n = 5) showed that both co-distribute in most focal adhesions (Fig. 7A–C; Pearson correlation coefficient: 0.79). Slight differences, however, were seen in EGF-induced active lamella. By time-lapse monitoring newly-appearing, paxillin-positive adhesion sites were initially negative for dSH2. After several minutes these sites acquired dSH2-positivity (Fig. 7A’, B’; Video S9). In contrast, comparison of the late focal adhesion marker zyxin with dSH2 (n = 5) did not reveal any visible differences in newly forming focal adhesion sites upon EGF treatment (not shown). But a differential distribution of both was discernible in larger, i.e. older sites. dSH2 fluorescence was preferentially localized to the more peripheral areas of these focal adhesions while zyxin was restricted to the more central parts (Fig. 8A, B). The line profiles of the recorded fluorescence in Fig. 8A’ further highlight the different distribution patterns. In addition, large and exclusively zyxin-positive structures were detected in the central part of cells (Fig. 8C, D) presumably representing fibrillar adhesions. When cells with such large zyxin-containing adhesions were treated with EGF, the central fibrillar adhesion sites disassembled to allow de novo formation of focal adhesions in the extending cell periphery (Fig. 8D, D’; Video S10).

## Discussion

**Figure 9:**
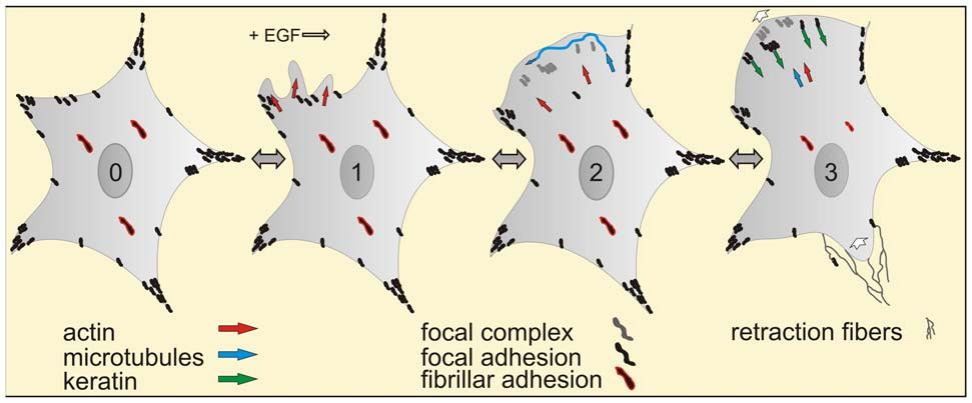
Schematic summary of early EGF effects on cell motility and cytoskeletal remodeling. Note that the depicted cascade is initiated by EGF but that each of the successive steps is reversible and subject to additional regulation. (0) shows a single cell in the absence of EGF with mature focal adhesions, that are positive for paxillin, dSH2 and zyxin, in the cell periphery in black and fibrillar adhesions in the central part of the cell, that contain little dSH2 but are enriched in zyxin (red-framed black streaks). (1) After addition of EGF actin-rich juxtamembranous regions (red arrows) form highly dynamic ruffles and lamellipodia that are devoid of keratin filaments, microtubules and focal adhesions. (2) Membrane domains with high ruffling activity develop lamella. Lamella formation is linked to the appearance of focal complexes that contain paxillin but are negative for dSH2 and zyxin (gray). Microtubule plus-ends enter these newly-formed cell extensions (blue arrows and wavy line). (3) Focal complexes mature into focal adhesions by recruitment of zyxin and tyrosine phosphorylation. The keratin network subsequently extends into the lamella by integration of keratin filament precursors (green arrows) that nucleate in the vicinity of focal adhesions and are transported toward the cell interior in an actin-dependant fashion. After disassembly of fibrillar adhesions the polarized cell then translocates toward the leading edge (direction of migration indicated by white arrows) leaving behind retraction fibers at its rear (thin lines).

Cellular motion has been dissected into repetitive cycles of biophysical processes, i.e. front edge extension by formation of lamellipodia and lamella, front adhesion, transcellular contraction and rear release [43]. The cycles are initiated by polarized cytoskeletal reorganization of sessile cells which transform from a stably-anchored epithelial state into a transiently-attached, fibroblastoid state to enable directed locomotion. We concentrated on these early steps of the migratory cascade that are under the direct regulatory control of EGF. The goal was to develop an integrated view of the early EGF-dependent cytoskeletal response by correlating alterations in the actin, microtubule and intermediate filament systems to each other and to the formation and maturation of focal adhesion sites. The emerging scenario is summarized in Fig. 9 as a series of sequential steps, each of which is reversible and entails distinct molecular factors and decisions.

Probably the most prominent and consistent effect of EGF treatment was the increase in ruffling, which started instantaneously upon growth factor addition and persisted up to 1 h. Ruffles are propagated along the cell surface. They are functionally and structurally related to active lamellipodia that appear in the cell periphery at the cell-ECM interface. Ruffling relies primarily on the actin system (red arrows in cell 1 of Fig. 9). It is likely independent of gene transcription but rather involves modification of pre-existing gene products and is induced by Rac1 involving phosphorylation of guanine nucleotide exchange factors (GEFs; [11], [44] and references therein). We established a comparatively simple way to evaluate the ruffling reaction in living cells meeting the periodic and wavelike nature of ruffling and thereby provide a possibility to quantitatively assess immediate EGF effects on cytoskeletal-dependent cell motility. This tool is of potential use for predicting and evaluating EGF responsiveness in patient-specific tumor therapy.

Laminar cell extensions that are attached to the extracellular matrix emerge from the cell body behind and under active lamellipodia [18], [45]. They are referred to as lamella [37], [39]. Microtubule ends were found to enter these regions (blue wavy line in cell 2 of Fig. 9). They appear to traverse in a random process the lamellar space and repeatedly bounce off the actin-rich membrane skeleton (Videos S4, S5). As far as we know, this probing activity has not been described to date. We consider it unlikely that mature focal adhesions are present at this stage, since the probing is different from the “search and capture” mode previously summarized [46], which relies on the presence of actin stress fibers and focal adhesions and occurs at a later stage of the migratory cascade.

Lamella formation is coupled to the appearance of focal complexes at the lamellipodial-lamella transition zone (grey streaks in cell 2 of Fig. 9). They are derived from nascent adhesions that are less than 0.25 µm in size and start to assemble in active lamellipodia [37], [39]. In the current study, enlarging paxillin-positive structures (>0.2 µm) were observed at the lamellipodial-lamellum junction ∼4 min after EGF addition. In molecular terms, it has been shown that EGFR signaling and focal adhesion dynamics are linked through the SRC-3 splicing isoform SRC-3C4 to the focal adhesion kinase FAK [22]. FAK is a multifunctional enzyme that promotes assembly of nascent adhesions by recruitment of talin [21] and affects actin dynamics through phosphorylation-dependent interaction with ARP2/3 and regulation of Rac and Rho activity by GEF/GAP localization [41], [47], [48]. Taken together, these molecular data support a tight cross talk between EGF signaling and focal adhesion formation.

In accordance with previous reports on other cell types [29] we observed that paxillin recruitment and tyrosine phosphorylation of paxillin and other adhesion proteins occur sequentially while focal complexes mature into 1–3 µm long and zyxin-positive focal adhesions (new black streaks in cell 3 of Fig. 9). Paxillin phosphorylation takes 2–5 min (Fig. 7; see also [29]) and is in large part mediated by EGF-induced Src-FAK signaling [49], [50], [51]. This further highlights the central functions of FAK in the EGF-dependent motility response. The relevance of FAK for cell motility is also underscored by the observation that increased FAK expression and autophosphorylation is associated with increased invasiveness of different tumors including gastric carcinomas ([52] and references therein).

We noted that large zyxin-positive fibrillar adhesions are disassembled in the presence of EGF while maturing focal adhesions increase (compare cells 2 and 3 in Fig. 9). This indicates that fibrillar adhesion-zyxin is recruited to newly-formed focal adhesion sites in preparation of cell translocation. The coincident acquisition of dSH2-positivity observed in this study suggests that zyxin-recruitment and tyrosine phosphorylation of focal adhesion components such as paxillin are a feature of focal adhesion maturation and not needed per se for focal complex formation as proposed in the model put forward by Gardel et al. [37]. Focal adhesion maturation is also coupled to their association with actin stress fibers and the development of myosin II-dependent tension [53]. In support, we observed incremental accumulation of actin stress fibers and enlarging focal adhesions. These stress fibers are not only of relevance for the mechanical, myosin-driven response but also serve as guidance cues for microtubule ends that regulate focal adhesion stability [40].

We observed that the keratin cytoskeleton is the last cytoskeletal filament system to enter lamella. The keratin network starts to extend into the lamella immediately after mature, dSH2-positive focal adhesions have formed (Video S6). It has been shown that focal adhesions favor nucleation of keratin filaments that are subsequently added to the peripheral cytoskeleton [24]. The newly-formed and continuously growing keratin particles are transported inward (green arrows in cell 3 of Fig. 9). This transport is to a large degree dependent on intact actin filaments and has been shown to occur along focal adhesion-anchored stress fibers [24], [54].

Later steps of the migratory response were not investigated in this study as they are far downstream of EGF signaling and involve a complex series of further alterations such as microtubule-dependent focal adhesion turnover, activation of the actin-myosin system (white arrows in cell 3 of Fig. 9), detachment of the cell rear and retraction fiber formation (thin wavy lines in cell 3 of Fig. 9) (reviews in [37], [40], [41].

The identified sequence of events associated with cytoskeletal reorganization will help to sort details of the underlying molecular mechanisms of the EGF response and to further unravel their multidimensional cross talk. Furthermore, it shall serve as a basis for understanding the resulting migratory behavior of cancer cells. This is an important prerequisite to model and to understand, how EGF-dependent cell motility contributes to tumor cell behavior and may thus help to identify specific tumors that are suitable targets for anti-EGF receptor-based therapies.

## Supporting Information

Video S1 **Ruffling activity is increased upon addition of EGF. MKN1 and Hs746T cells were transfected to produce enhanced yellow fluorescence protein (YFP) under CMV-promoter control.** The fluorescence was recorded by confocal laser scanning microscopy three times per minute in the absence of EGF and after addition of 5 ng/ml EGF (corresponding Fig. 1A–B’). Note the EGF-induced cell spreading and the appearance of multiple ruffles and lamellipodia that leads to extensive lamella formation in MKN1 cells but not in Hs746T cells.
(MPG)

Video S2 **EGF-induced restructuring of the actin and keratin cytoskeleton.** The overlay images show a MKN1 cell that is labeled with mRFP-actin (red) and HK18-YFP (green) before and after addition of 5 ng/ml EGF (see also corresponding Fig. 2D, D’). Fluorescence images were recorded by confocal laser scanning microscopy (2 frames/min). Note the appearance of multiple actin-rich and keratin-free ruffles upon EGF treatment.
(MPG)

Video S3 **EGF does not affect microtubule plus-end-dynamics.** Microtubule plus-ends were labeled by EB3-CFP in a MKN1 cell. The time-lapse sequence (10 frames/min) of fluorescence images (inverse presentation; left) and corresponding DIC images (right) was recorded first in the absence of EGF and then in the presence of 30 ng/ml EGF (corresponding Figure 3A, B). Note that EGF induces extensive ruffling and cell translocation that is coupled to retraction fiber formation at the rear. The associated substantial cell shape alterations necessitated adjustment of the focal plane resulting in a few out of focus images. Note also that plus-end-dynamics are not altered by EGF treatment.
(MPG)

Video S4 **Microtubule exploration of EGF-induced cell extension.** Fluorescence microscopy (inverse presentation) of a peripheral segment of a MKN1 cell synthesizing EB3-CFP after addition of 100 ng/ml EGF. The recording (6 frames/min) begins 1.5 min after EGF addition (see also corresponding Fig. 3E). Note the exploratory extension of microtubules into the newly-formed cell extension. Bar, 5 µm.
(MPG)

Video S5 **Triple labeling of MKN1 cells reveals sequential reorganization of cytoskeletal filaments in response to EGF.** MKN1 were transfected with expression constructs for mRFP-actin, EB3-CFP and HK18-YFP. The composite Video displays images that were recorded every 38 s starting 10 min after addition of 30 ng/ml EGF. It depicts the individual fluorescence patterns of EB3-CFP (EB3), mRFP-actin (ACTB) and HK18-YFP (KRT18) first in inverse presentation separately (cell contours demarcated in orange), then as a merged overlays of EB3-CFP (false green color) and mRFP-actin (red), and finally as merged overlays of EB3-CFP (false green color), mRFP-actin (red) and HK18-YFP (false blue color) as indicated. Note that EGF-induced cell extensions are positive for actin and that dynamic microtubules enter these regions which contain very little keratin. Bar, 10 µm.
(MPG)

Video S6 **EGF-induced restructuring of the actin (left) and keratin cytoskeleton (right) in relation to focal adhesion dynamics (middle).** The images were recorded in a MKN1 cell (see corresponding Fig. 5) that was labeled with mRFP-actin (left), CFP-dSH2 (middle) and HK18-YFP (right). The images (inverse presentation) were collected at 2 frames/min before and after addition of 5 ng/ml EGF. Note the appearance of actin-rich ruffles that either lead to lamella formation (at left cell margin) with dSH2-labeled focal adhesions and extending keratin filaments or disappear (at right cell margin).
(MPG)

Video S7 **Lamella differ from ruffles and lamellipodia by the presence of focal adhesions.** The time lapse series (25 s recording intervals) shows overlays of DIC recordings and CFP-dSH2 fluorescence (false red color) in MKN1 cells (see corresponding Fig. 6 A, A’). Note the induction of abundant, dSH2-negative ruffles and lamellipodia upon addition of 5 ng/ml EGF.
(MPG)

Video S8 **Focal adhesions are formed and turn over in lamella (corresponding** **Fig. 6** **B, B’).** The micrographs show overlays of YFP (white) and paxillin-dsRed fluorescence in a MKN1 cell before and after addition of EGF (5 ng/ml). Images were recorded every 28 s.
(MPG)

Video S9 **Paxillin is recruited to focal adhesions prior to tyrosine phosphorylation in newly-formed lamella upon EGF treatment (see also** **Fig. 7** **).** The images are taken from a MKN1 cell simultaneously expressing paxillin-dsRed and CFP-dSH2 (false green color) immediately after addition of 5 ng/ml EGF. The composite Video presents the overlays of paxillin-dsRed and CFP-dSH2 (top) and the overlays in combination with DIC micrographs (bottom). Images were recorded every 25 s by confocal laser scanning microscopy. Note the appearance of paxillin-positive focal adhesion sites in the proceding lamellum prior to dSH2 recruitment.
(MPG)

Video S10 **Zyxin partially co-distributes with dSH2 in focal adhesions and is recruited from zyxin-enriched fibrillar adhesion upon EGF treatment.** MKN1 were co-transfected with constructs encoding RFP-zyxin and CFP-dSH2 (depicted in false green color). Note that both co-localize in focal adhesions in the cell periphery but that the more centrally located focal adhesions and large fibrillar adhesions are predominantly positive for zyxin and contain very little dSH2 label. The video depicts a cell before and after addition of 5 ng/ml EGF (recording interval 50 s; see also corresponding Fig. 8D, D’). Note the reduction of the very large zyxin-positive plaque-like areas in the cell center and the appearance of novel contacts in newly-formed lamella (e.g., lower right). Bar, 5 µm.
(MPG)
